# Development of Adsorptive Membranes for Selective Removal of Contaminants in Water

**DOI:** 10.3390/polym14153146

**Published:** 2022-08-02

**Authors:** Priyalatha M. Kirisenage, Syed M. Zulqarnain, Jordan L. Myers, Bradley D. Fahlman, Anja Mueller, Itzel Marquez

**Affiliations:** 1 Department of Chemistry and Biochemistry, Central Michigan University, Mount Pleasant, MI 48859, USA; madhu1kp@cmich.edu (P.M.K.); myers5jl@cmich.edu (J.L.M.); fahlm1b@cmich.edu (B.D.F.); muell1a@cmich.edu (A.M.); 2 School of Engineering and Technology, Central Michigan University, Mount Pleasant, MI 48859, USA; zulqa1sm@cmich.edu

**Keywords:** imprinting polymerization, graphitic carbon nitride, adsorbents, adsorptive membranes, water treatment, arsenate, ammonia

## Abstract

The presence of arsenic and ammonia in ground and surface waters has resulted in severe adverse effects to human health and the environment. Removal technologies for these contaminants include adsorption and membrane processes. However, materials with high selectivity and pressure stability still need to be developed. In this work, adsorbents and adsorptive membranes were prepared using nanostructured graphitic carbon nitride decorated with molecularly imprinted acrylate polymers templated for arsenate and ammonia. The developed adsorbent removed arsenate at a capacity and selectivity similar to commercial ion-exchange resins. Ammonia was removed at higher capacity than commercial ion exchange resins, but the adsorbent showed lower selectivity. Additionally, the prepared membranes removed more arsenate and ammonia than non-imprinted controls, even in competition with abundant ions in water. Further optimization is required to improve pressure stability and selectivity.

## 1. Introduction

The presence of toxic ions and nutrients in ground and surface waters has resulted in severe adverse effects to human health and the environment [1]. Among these ions, arsenic (As) contamination in groundwater is considered as one of the major problems in the world. Chronic exposure to arsenic causes adverse effects on human health such as damage to the gastrointestinal tract, respiratory, skin, liver, nervous system, and cardiovascular diseases, and even diabetes [2,3]. High arsenic concentrations have been reported in Taiwan, Chile, India, Mexico, Argentina, Bangladesh, several areas in USA, New Zealand, Sri Lanka, Canada, Japan, Poland, and China, among others [4]. Currently, it is estimated that 200 million people around the world could be exposed to high levels of arsenic [3]. 

Other than naturally occurring arsenic species, anthropogenic sources are also contributors to groundwater contamination. Arsenic is used in insecticides, herbicides, food additives and medicinal preparations [5].

Arsenic can be found in different environments (air, soil, water, living organisms) in different oxidation states; arsenate, As(V), arsenite, As(III), arsenic, As(0), and arsenide, As(-III). Among them, arsenite and arsenate are the most common and the most toxic species found in groundwater. Conventional treatment of As(III) in water consists of oxidation to As(V) under aerobic conditions and at a pH of above 7, and then removal of As(V). Removal of As(V) is conventionally done by filtration, adsorption, ion exchange, and membranes [6,7]. In recent years, novel materials for the removal of arsenic have been developed, including graphite oxides, carbon nanotubes, metal organic structures, and magnetic nano composites [5,7].

Another compound with severe adverse effects in water is the common nutrient ammonia. Ammonia is soluble in water and is present in varying concentrations in ground and surface waters. The presence of ammonia in water is undesirable for several reasons. Ammonia is a nitrogen source and nutrient for algae and plant life, contributing to eutrophication of lakes and rivers, the depletion of dissolved oxygen, and toxicity in fish and other aquatic animals [8]. A common source of ammonia is agricultural runoff, which easily moves into underground aquifers. 

Ammonia exists in two forms in water, as ammonium ion (NH_4_^+^) and ammonia (NH_3_), depending on the pH. NH_3_ is more toxic than charged ammonium ions because it is a neutral molecule, and thus can diffuse easily across epithelial membranes. Ammonia also consumes chlorine, resulting in reduced efficiency of common water purification techniques such as chlorination.

Ammonia and ammonium ions can be removed from wastewater using biological denitrification, stripping, ion exchange, break-point chlorination, and chemical precipitation [9]. More recent methods include photocatalysis and electrochemical oxidation [10].

Among the removal methods for As(V) and ammonia, adsorption and membrane processes present unique advantages. For instance, membrane processes have shown 95% or more pollutant rejection in optimal conditions [11,12]. However, pollutant rejection differs greatly depending on membrane and operational conditions such as membrane pore size and surface properties [11]. On the other hand, adsorption processes are considered simple to operate and to design, environmentally benign and require low energy cost. Nevertheless, commonly used adsorbents, such as activated carbon, bind contaminants weakly and are not selective. 

Adsorptive membranes have become an effective way to remove contaminant ions and nutrients from water [9]. When compared with conventional membranes, adsorptive membranes provide high retaining efficiency for pollutants, low energy consumption and high permeate flux. Pollutant removal with adsorptive membranes can also be much faster than with conventional adsorbents because the contaminants can be brought to the external and internal binding sites by convective flow in the adsorptive membrane systems, rather than by slow external or internal diffusions in adsorption systems [13]. However, the typical adsorptive membranes, composed of natural or synthetic polymeric membranes, present low chemical and thermal stability, uncontrollable pore size, and a trade-off between permeability and selectivity [9]. 

Although many polymeric and inorganic membranes have been explored over the past years [14,15], it is still an enormous challenge to attain a single membrane that meets all the requirements of high permeation flux, high selectivity, high pressure stability, and antifouling ability. Some of these challenges can be overcome by using molecularly imprinted polymers (MIPs), which facilitate selective removal. MIPs have been used to remove a wide variety of contaminants from water, from contaminants of emerging concern [16], to heavy metal ions [17,18,19], to radioactive materials [20]. For instance, Gornik et al. [21] investigated MIPs as sorbents for the removal of antidepressants from wastewater. Their materials were stable, reusable, and showed higher sorption capability than activated carbon.

MIPs have shown promising results for the removal of arsenate and other contaminants from water [1,17,22,23,24]. However, when used in pressurized and varying pH environments, MIPs can lack stability and the necessary strength and surface area needed for large scale operation [16,25]. Graphitic carbon nitride (g-C_3_N_4_) is a promising scaffold for constructing adsorptive membranes, due to its facile synthesis, chemical versatility, intrinsic porosity, high strength, and natural abundance [26]. Substituting carbon by nitrogen creates new functionalities, such as catalytic activity, as well as improved separation and self-cleaning [27]. Graphitic carbon nitride also provides advantages such as robust mechanical properties, substantial surface area, non-toxicity, and facile synthesis from readily available precursors [26,28,29]. 

Graphitic carbon nitride has been used for several applications, including drug delivery systems [30], photocatalytic hydrogen generation, supercapacitors, and disinfection [31,32]. Furthermore, due to its intrinsic porous characteristics and high stability, g-C_3_N_4_ has been used to fabricate functional membranes for applications in water treatment (desalination and contaminants removal), gas separation and pervaporation [26]. Table 1 summarizes the characteristics of commercial polymeric and ceramic membranes compared to g-C_3_N_4_ membranes.

Despite its advantages, g-C_3_N_4_ presents structural disorder and poor dispersibility [42]. To improve g-C_3_N_4_ properties, the combination with polymers has provided promising results. Examples of g-C_3_N_4_-polymer materials include g-C_3_N_4_ as a photoinitiator for polymer synthesis [43,44,45], polymer-modified g-C_3_N_4_ for improved dispersibility, and g-C_3_N_4_ hydrogels [42]. In this work, nanostructured g-C_3_N_4_ decorated with acrylate MIPs templated for arsenate and ammonia was synthesized to produce adsorbents and adsorptive membranes for the removal of arsenate and ammonia from water. Compound removal and selective adsorptivity were tested in adsorption resins and membranes. This work serves as a proof-of-concept for novel materials in water treatment.

## 2. Materials and Methods

### 2.1. Materials

The following materials were obtained and used as received from Sigma Aldrich (St. Louis, MO, USA): methacrylic acid (99%), methacrylamide (98%), methyl methacrylate (98%), 2,2′-Azobis(2-methylpropion-amidine) dihydrochloride (AAPD) (97%), ethylene glycol dimethacrylate (EDMA) (98%), sodium arsenate (98%), ammonium hydroxide (28%), urea (99–100%), and activated charcoal (99.997%, 100 mesh size). Ion exchange resins (Ambersep 21K and Ambersep G-26-H) were obtained from Dupont (Wilmington, DE, USA). Deionized ultra-filtered (DIUF) water was obtained from an E-Pure water purification system (Barnstead E-Pure D4641, Dubuque, IA, USA) and was collected at 18 M Ohm. Hydrochloric acid (HCl) (12 M) was obtained from Fisher Scientific (Waltham, MA, USA). 

### 2.2. Synthesis of Graphitic Carbon Nitride 

Pristine g-C_3_N_4_ was synthesized using 20 g of urea placed in a covered crucible and heated at 550 °C for 4 h at a heating rate of 2.5 °C/min in a muffle furnace (Lindberg 51894, Waltham, MA, USA).

### 2.3. Synthesis of Porous Arsenate-Imprinted and Non-Imprinted 30:70 Methacrylic Acid: Methacrylamide Polymer

Methacrylamide (9.0140 g, 0.106 mol, 0.7 eq) and methacrylic acid (4.00 mL, 0.047 mol, 0.3 eq) were added to a flat-bottom reaction vessel and dissolved in 150 mL DIUF water. HCl (0.1 M, 10.0 mL) was added to the solution to quaternize the amide of the methacrylamide. Nitrogen was bubbled through the solution during the reaction to increase the surface area of the resulting polymer. 

For the imprinted polymer only, the template sodium arsenate (Na_2_HAsO_4_) (0.05 g, 0.0002 mol, 0.001 eq) was then added. Thereafter, 13.64 µL (7 × 10^−5^ mol, 0.0005 eq) of the crosslinking agent EDMA was added, followed by the initiator AAPD (0.0443 g, 0.0002 mol, 0.001 eq). The reaction was stirred in an UV reactor (Rayonet RPR-100, Branford, CT, USA) for 4 h. The product was filtered using a Buchner funnel and lyophilized (Labconco FreeZone, Kansas City, MO, USA) to remove water. The template was removed by dialysis with brine solution (1 M). Yield: imprinted 90%, non-imprinted 86.2%.

### 2.4. Synthesis of Porous Ammonia-Imprinted and Non-Imprinted 70:30 Methacrylic Acid: Methacrylamide Polymer

Methacrylamide (3.061 g, 0.035 mol, 0.3 eq) and methacrylic acid (7.046 mL, 0.07 mol, 0.7 eq) were added to a flat-bottom reaction vessel and dissolved in 100 mL DIUF water. Nitrogen was bubbled through the solution during the reaction to increase the surface area of the resulting polymer. For the imprinted polymer only, the template ammonium chloride (0.6308 g, 0.01 mol, 0.1 eq) was then added. The crosslinker EDMA (13.64 µL, 7 × 10^−5^ mol, 0.0005 eq) was added. Lastly, AAPD (0.0443 g, 0.0002 mol, 0.001 eq) was added to initiate the polymerization. The reaction was stirred in an UV reactor for 4 h. The product was filtered using a Buchner funnel and lyophilized to remove water. The template was removed by dialysis with DIUF water. Yield: imprinted 93%, non-imprinted 93%.

### 2.5. Synthesis of Porous Arsenate-Imprinted and Non-Imprinted Polymer with Graphitic Carbon Nitride

Graphitic carbon nitride (1.0000 g) was added to a flat-bottom reaction vessel and dissolved in 75.0 mL of DIUF water and 75.0 mL of acetonitrile. Then, methacrylamide (9.016 g, 0.106 mol, 0.7 eq) and methacrylic acid (4.00 mL, 0.047 mol, 0.3 eq) were added. Thereafter, 5.77 µL (3.059 × 10^−5^ mol, 0.0002 eq) of the crosslinking agent (EDMA) was added. After that, HCl (0.1 M, 10.00 mL) was added to the solution to quaternize the amide group of the methacrylamide. The reaction vessel was placed on the stirrer under two blue light sources (GloGlow E27 18 W LED 460 nm, Shenzhen, China) for 48 h. Nitrogen was bubbled through the solution during the reaction to increase the surface area of the resulting polymer. For the imprinted polymer only, the template sodium arsenate (Na_2_HAsO_4_) (0.05 g, 0.0002 mol, 0.001 eq) was added. The product was filtered using a Buchner funnel and dried. The template was removed by dialysis with DIUF water. Yield: imprinted 85.4%, non-imprinted 86.2%.

### 2.6. Synthesis of Porous Ammonia-Imprinted and Non-Imprinted Polymer with Graphitic Carbon Nitride 

Graphitic carbon nitride (1.0000 g) was added to a flat-bottom reaction vessel and dissolved in 75.0 mL of DIUF water and 75.0 mL of acetonitrile. Then, methacrylamide (3.061 g, 0.035 mol, 0.3 eq) and methacrylic acid (7.046 mL, 0.081 mol, 0.7 eq) were added. Thereafter, 13.64 µL (7 × 10^−5^ mol, 0.0005 eq) of the crosslinking agent (EDMA) was added. The reaction vessel was placed on the stirrer under two blue light sources (GloGlow E27 18 W LED 460 nm) for 48 h. Nitrogen was bubbled through the solution during the reaction to increase the surface area of the resulting polymer. For the imprinted polymer only, the template ammonium chloride (0.6283 g, 0.01 mol, 0.1 eq) was added. The product was filtered using a Buchner funnel and dried. The template was removed by dialysis with DIUF water. Yield: imprinted 78%, non-imprinted 75%.

### 2.7. Adsorptive Membrane Synthesis

Graphitic carbon nitride (1.0000 g) was added to a flat-bottom reaction vessel and dissolved in 22.5 mL of DIUF water and 22.5 mL of acetonitrile. Then, for arsenate, methacrylamide (5.4096 g, 0.6356 mol, 0.7 eq) and methacrylic acid (2.40 mL, 0.024 mol, 0.3 eq) were added. For ammonia, methacrylamide (1.836 g, 0.0211 mol, 0.3 eq) and methacrylic acid (4.144 mL, 0.049 mol, 0.7 eq) were added. Thereafter, 3.46 µL (1.8 × 10^−5^ mol, 0.0002 eq) of the crosslinking agent (EDMA) was added. For the arsenate non-imprinted and imprinted membranes only, HCl (0.1 M, 6.00 mL) was added to the solution to quaternize the amide group of the methacrylamide. The mixture was sonicated for 30 min. The reaction vessel was placed on the stirrer under two blue light sources (GloGlow E27 18 W LED 460 nm) for 2 h. Nitrogen was bubbled through the solution during the reaction to increase the surface area of the resulting polymer. For the imprinted polymer only, the template sodium arsenate (Na_2_HAsO_4_) (0.03 g, 0.0001 mol, 0.001 eq) or ammonium chloride (0.3786 g, 0.01 mol, 0.1 eq) was then added. The product was filtered using a Buchner funnel and dried for 2 h under the blue light. Then the resulting product was kept in the refrigerator for 20 min. The template was removed by washing with DIUF water under reduced pressure (vacuum).

### 2.8. Adsorption Column Removal Experiments 

The synthesized materials were tested for ammonia and arsenate removal. As controls, graphitic carbon nitride and non-imprinted polymers were also tested. Activated carbon and ion exchange resins were used for removal comparisons. Ammonia and arsenate-imprinted polymers were tested under the same conditions. Polymers were crushed using mortar and pestle to a 250 μm particle size, unless otherwise specified. Polymers were swollen in DIUF water (20.0 mL) for 24 h before use. All other materials were used as received or prepared. To pack the columns (6″ length × 0.35″ diameter), 200 mg of each material were used. Known concentrations of sodium arsenate (5, 10 and 20 mg/L, 20.0 mL) or ammonium chloride (2, 4 and 8 mg/L, 20.0 mL) were added to the columns. For competition experiments, a 50%:50% sodium arsenate: sodium chloride solution (20 mg/L, 20.0 mL) was used for arsenate. For ammonia, a 50%:50% ammonium chloride: calcium ion (Ca^2+^) solution (8 mg/L, 20 mL) was used. Eluent was collected for analysis. Each experiment was run in triplicate. 

### 2.9. Adsorptive Membrane Removal Experiments

A known concentration of sodium arsenate (20 mg/L, 20.0 mL) or ammonium chloride (8 mg/L, 20 mL) was passed through the non-imprinted and imprinted membranes. For competition experiments, a 50%:50% sodium arsenate: sodium chloride solution (20 ppm, 20.0 mL) or a 50%:50% ammonia: calcium ion (Ca^2+^) solution (8 mg/L, 20 mL) was used. Filtrate was collected for analysis. All experiments were run in triplicate.

### 2.10. Analytical Measurements

Chemical composition of the materials was characterized by Fourier-transform infrared spectroscopy (FT-IR, Thermo Electron Corp. Nicolet 380, Waltham, MA, USA), and morphology by scanning electron microscopy (SEM, Hitachi S-3400N, Chiyoda City, Tokyo, Japan). Prior to SEM imaging, samples were sputter-coated with a 60:40 mixture of gold:palladium to obtain contrast using a Hummer 6.2 sputter coater. The elemental distribution of the materials was mapped by energy-dispersive X-ray spectroscopy (EDS, Hitachi S-3400N, Chiyoda City, Tokyo, Japan)

Ammonium and calcium ion concentrations were measured using ion chromatography (Thermo Scientific Dionex Aquion IC System, Waltham, MA, USA, cation eluent 20 mM, methanesulfonic acid flow rate 0.5 mL/min, suppressor current 30 mA). For arsenate, the column eluent was collected and HNO_3_ (2%) was added to the solution. Arsenate concentrations were measured using inductively coupled plasma-optical emission spectrometry (ICP-OES, Agilent 5800, Santa Clara, CA, USA, wavelength 188.980 nm, pump speed 12 rpm, and plasma flow rate 12.0 L/min). Chloride concentration was measured using a chloride ion selective electrode (Accumet pH meter 25, Westford, MA, USA). 

## 3. Results

The lack of selectivity of conventional adsorptive and membrane materials reduces their effectiveness for the removal of a variety of contaminants. In this work, the combination of nanostructured g-C_3_N_4_ with acrylate MIPs templated for arsenate and ammonia were synthesized to produce adsorbents and adsorptive membranes that are arsenate- and ammonia-selective and pressure-stable. As a proof of concept, non-imprinted and imprinted polymers and membranes were synthesized and tested. Commercially available activated carbon and ion exchange resins were tested for comparison.

### 3.1. Materials Synthesis and Characterization

Acrylates are considered inexpensive materials and have been used for water treatment materials [46,47]. They are commonly used for imprinted polymers for water contaminants [23]. In this work, acrylates were used for the imprinted polymerization of arsenate and ammonia to increase the selectivity of the materials. The ratio between acrylic acid and acrylamide for imprinting polymerization for heavy metal ions has been developed over the years [17,22]. Graphitic carbon nitride was added to the materials to increase pressure stability and enable future photocatalysis applications [48,49,50].

**Figure 1:**
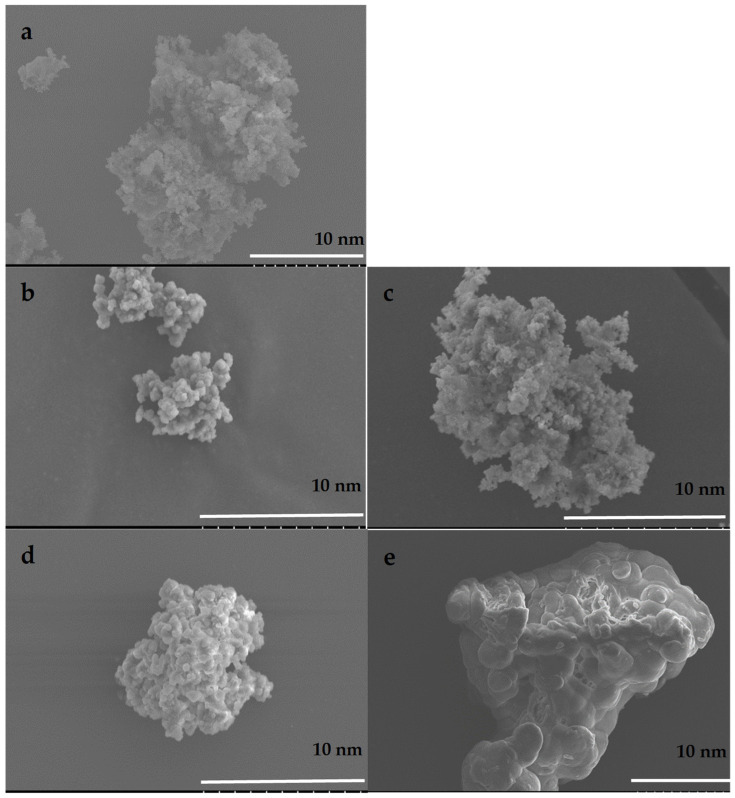
SEM images for (**a**) g-C_3_N_4_, (**b**) arsenate-imprinted polymer, (**c**) arsenate-imprinted polymer with g-C_3_N_4_, (**d**) ammonia-imprinted polymer, (**e**) ammonia-imprinted polymer with g-C_3_N_4_.

The yield of the polymers were all above 90%, showing that the polymerization was effective. However, the addition of g-C_3_N_4_ reduced the yield as low as 75%, probably due to differences in solubility between g-C_3_N_4_ and polymer. When added to the polymerization, g-C_3_N_4_ also acted as an initiator, therefore the initiator was removed from this polymerization. It was also observed that g-C_3_N_4_ acted as a crosslinker. Therefore, the amount of crosslinker was reduced in the polymerization with g-C_3_N_4_. Figure 1 shows SEM images of the starting materials and their combination. The images show a homogenous composition of the components in both non-imprinted and imprinted materials with similar particle sizes. Occasional agglomeration is also observed in the ammonia-imprinted samples.

**Figure 2:**
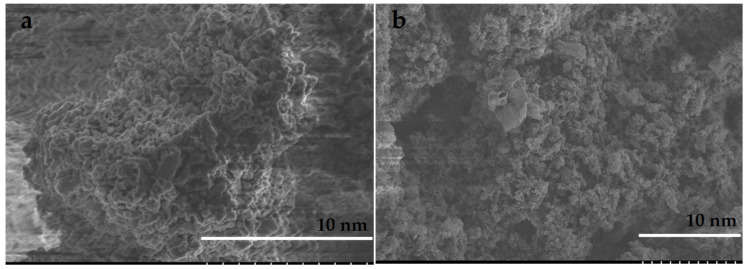
SEM images for (**a**) arsenate-imprinted membrane, (**b**) ammonia-imprinted membrane.

Membranes were prepared by gravity filtration [51]. In short, the monomers were oligomerized in a high concentration solution by blue light (460 nm) for 2 h before they were polymerized in the filtration setup. Figure 2 shows SEM images of the membranes. Images show a homogeneous distribution of the components with a high degree of surface roughness. Images for non-imprinted materials are shown in Supplementary Materials Figures S1 and S2.

**Figure 3:**
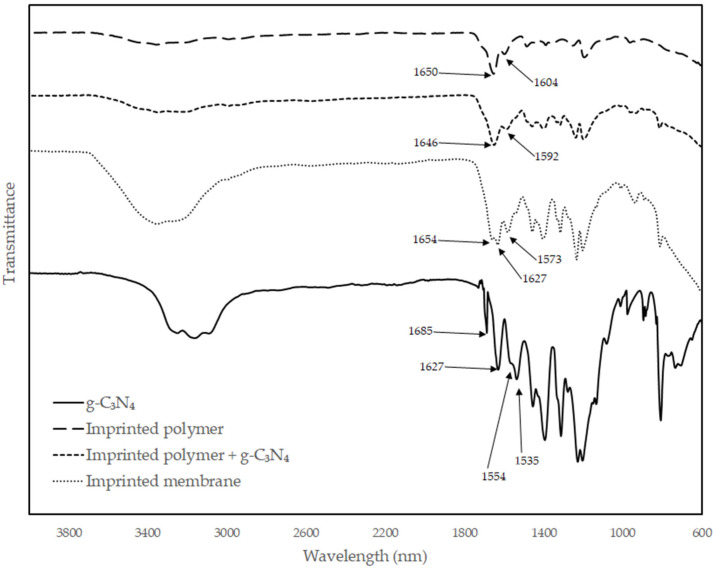
FT-IR for g-C_3_N_4_, imprinted adsorbents and membranes for arsenate removal.

**Figure 4:**
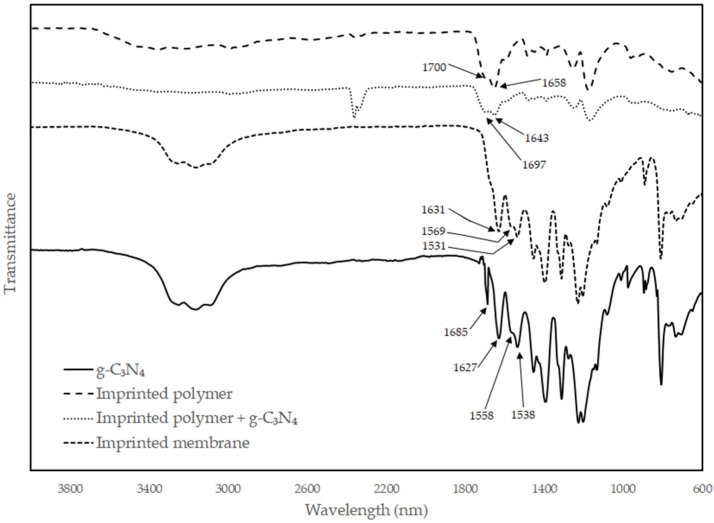
FT-IR for g-C_3_N_4_, imprinted adsorbent and membrane for ammonia removal.

FT-IR characterization of materials is shown in Figure 3 and Figure 4. Results show the presence of all expected functional groups. In these compounds, the carbonyl region (1750 to 1600 cm^−1^) overlapped with the C=C and C=N region (1700 and 1550 cm^−1^). The carboxylic acid peak of the methacrylate shifted to lower values when deprotonated to the anion, as well as with different amounts of hydrogen bonding [25]. Forming dimers with itself or an amide, the carboxylic acid peak shifted to higher values. The ratio between the acid and amide peak was also affected by interaction with neighboring compounds. The amide, being slightly more hydrophobic than the acrylate, moved towards the more hydrophobic g-C_3_N_4_, reducing the amide peak in comparison to the acrylate peak. For the arsenate, an OH peak of the acrylic acid was observed at 3000–3500 cm^−1^. For g-C_3_N_4_, 1600 cm^−1^ (C=N), 1255–1428 cm^−1^ (C-N), and 809 cm^−1^ (CN-heterocycle) are the characteristic peaks.

### 3.2. Removal of Arsenate in Adsorption Columns

**Figure 5:**
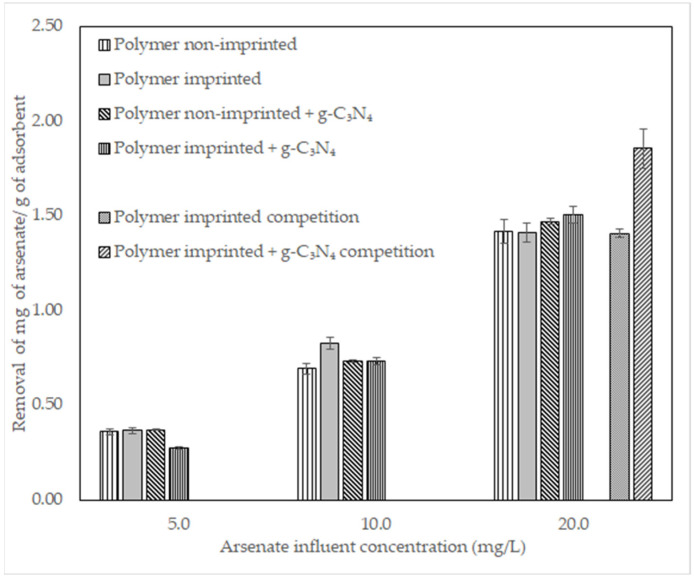
Removal of arsenate in columns by specified materials at different arsenate concentrations in the influent. Imprinted polymer with g-C_3_N_4_ outperforms all materials in competition experiment at the highest concentration, showing the selectivity of the material.

Removal of arsenate in adsorption columns with different materials is presented in Figure 5 as mg of arsenate adsorbed per g of adsorbent. Competition experiments were performed with a 50%:50% sodium arsenate: sodium chloride solution (20 mg/L). 

### 3.3. Removal of Ammonia in Adsorption Columns

**Figure 6:**
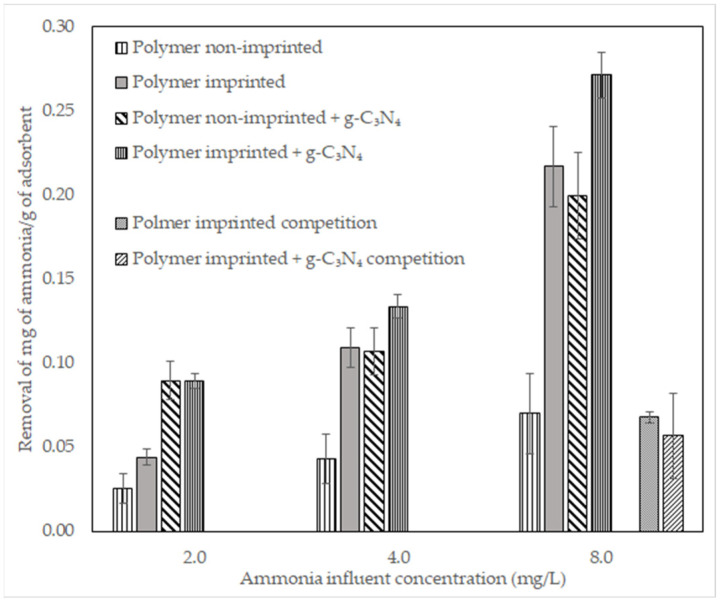
Removal of ammonia in columns by specified materials at different ammonia concentrations in the influent. Imprinted polymer with g-C_3_N_4_ performs similarly to other materials but competition experiments show no selectivity.

Removal of ammonia in adsorption columns with different materials is presented in Figure 6 as mg of ammonia adsorbed per g of adsorbent. Competition experiments were performed with a 50%:50% ammonia: calcium ion (Ca^2+^) solution (8 mg/L).

### 3.4. Comparison to Conventional Adsorbents

**Figure 7:**
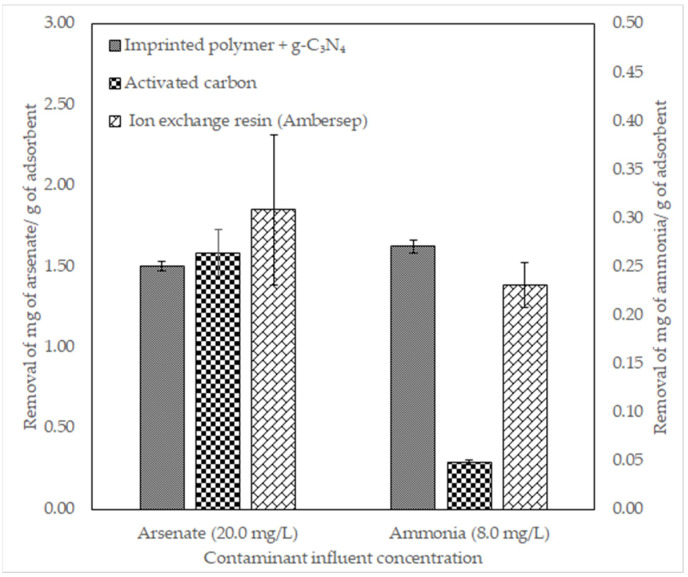
Arsenate and ammonia removal comparison between imprinted polymer with g-C_3_N_4_ and commercial adsorbents. The material developed removes arsenate similarly to activated carbon and ion exchange resin Ambersep 21 K. The removal of ammonia is greater than activated carbon and ion exchange resin Ambersep G-26-H.

Synthesized materials were compared to activated carbon and ion exchange resins. Ion exchange resin Ambersep 21 K was used to remove arsenate. Ambersep G-26-H was used to remove ammonia. Results are shown in Figure 7.

### 3.5. Initial Membrane Removal Results

**Figure 8:**
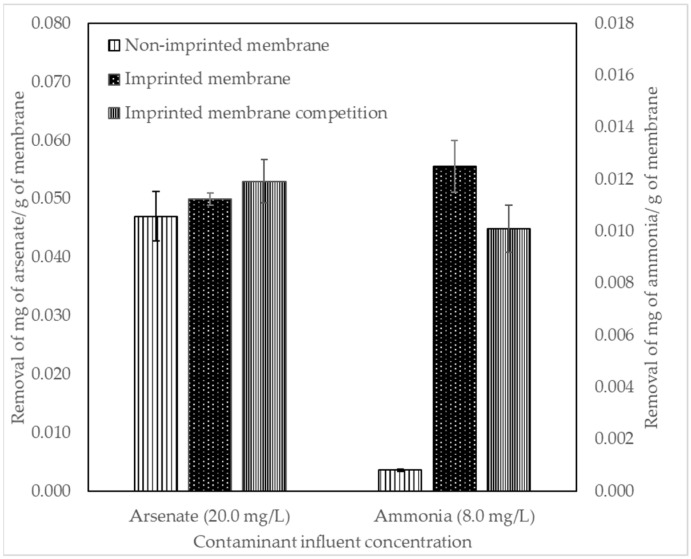
Arsenate and ammonia removal with non-imprinted and imprinted membranes and their respective competitions. The arsenate-imprinted membrane removes more arsenate in competition filtration. The ammonia-imprinted membrane removes more ammonia than the non-imprinted membrane, but it is not highly selective to ammonia.

Removal of arsenate and ammonia was tested in the prepared membranes. Results are shown in Figure 8.

## 4. Discussion

### 4.1. Materials Synthesis and Characterization

The polymerization method was based on earlier work described by Randhawa et al. [23]. The polymer is negatively charged due to the presence of carboxylic acid groups. It was used as is for the removal of the positively charged ammonium ions. For the removal of arsenate, the polymer was quaternized with acid to result in an overall positive charge. To increase the porosity of the polymer, nitrogen was bubbled through the solution during polymerization.

Polymerization with g-C_3_N_4_ required adjustments in the synthesis. Graphitic carbon nitride can act as initiator for acrylate polymerizations [45,46,48]. Therefore, in the combined polymerization, the initiator was removed. Furthermore, since g-C_3_N_4_ has been proven to be a visible-light-driven photoinitiator [45], the wavelength used for polymerization was changed to blue light (460 nm), resulting in a slower polymerization reaction. In addition, when polymerizing with g-C_3_N_4_, it was necessary to add a co-solvent, acetonitrile, to solubilize the g-C_3_N_4_ in the aqueous solution. Finally, when using the conventional amount of crosslinker, the imprinted molecule could not be removed, indicating that the g-C_3_N_4_ acted also as a crosslinker in the polymerization. Therefore, the amount of crosslinker was reduced in the polymerization solution with g-C_3_N_4_.

The membrane preparation also required adjustments. The filtration method was chosen for initial membrane preparation due to its simplicity and low cost. However, with a slower polymerization under blue light, it was not possible to fully polymerize the membrane during the time of filtration. Therefore, the mixture was oligomerized first under blue light (460 nm). Then, the reaction was completed during filtration.

The polymerizations were complete according to the yields observed, and the FT-IR characterization showed all the expected peaks for the polymers and g-C_3_N_4_. SEM images demonstrated that the materials are homogeneous and not phase-separated. This is illustrated by the absence of g-C_3_N_4_ sheets in the images and a consistent particle size and membrane surface roughness. The starting materials of the synthesis reported here are inexpensive and the materials and membranes are easy to prepare and easy to adapt to a variety of compounds.

### 4.2. Removal of Arsenate in Adsorption Columns

Results in Figure 5 show no significant difference in the removal of arsenate between the non-imprinted polymer and the arsenate-imprinted polymer. This is likely due to the limited number of imprinted sites, as the EDS mapping of the polymer before removing the template shows in Supplementary Materials (Figure S3). However, the results of competition experiments between arsenate and chloride demonstrated that arsenate bound more strongly to the adsorbent than chloride (1.857 and 0.138 mg/g, respectively) due to the imprinted sites, resulting in high selectivity of the imprinted polymer. Results also show that g-C_3_N_4_ does not significantly change the amount of arsenate removed. A summary of results is shown in Table 2.

While not directly comparable, other studies have reported total capacities at optimized conditions of 5.24 mg/g [52] and 106.3 mg/g [36] of arsenite in imprinted polymers, although not arsenate. The materials reported have been optimized for pH, time, and sorbent dose. Jagirani et al. [36] also evaluated competitive adsorption with several other ions, showing selectivity coefficients between 1.781 with nitrate ions (NO_3_^−^) and 2.590 with sulfate ions (SO_4_^2−^). However, arsenite and chloride ion competition was not reported. Gao et al. [53] prepared an arsenate MIP with 2-methacryloyloxyethyl-trimethyl ammonium chloride and silicon dioxide (SiO_2_). The maximum adsorption was 25.38 mg of arsenate per gram of adsorbent at optimum pH. The selectivity coefficients for arsenate were 8.814 and 7.898 relative to chromate and nitrate ions, respectively.

The materials in this work have not yet been optimized for pH, contact time, sorbent dose or other variables. Optimization of these variables and maximum adsorption would allow comparison of adsorption capacities with other reports. However, selectivity results were consistent with results from others [36,53]. To the best of our knowledge, the combination of MIPs with g-C_3_N_4_ has not been reported for arsenate removal.

### 4.3. Removal of Ammonia in Adsorption Columns

Results in Figure 6 show that the ammonia-imprinted polymer with g-C_3_N_4_ adsorbed a greater mass of ammonia when compared to the other materials tested. However, in competition with calcium ions, the removal of ammonia decreased (Table 3). Calcium ions have a higher charge density than ammonium ions, resulting in stronger binding to the negatively charged polymer. Additionally, the ring structure of g-C_3_N_4_ efficiently binds positively charged ions [54,55,56]. The combination of these two effects makes the ammonia-imprinted polymer with g-C_3_N_4_ highly effective in removing any positively charged ions, reducing selectivity. In spite of this, due to the imprinted sites, the material still removes ammonium ions. Adjustments to the synthesis need to be made to increase selectivity, including increasing the number of imprinted sites. 

Han et al. [57] reported ammonia adsorption by an MIP, although in gases and not water. The adsorbent was polymerized in organic solvent using a single monomer, unlike the work presented here. The material was then optimized for synthesis pH, pH solution ratio and crosslinker ratio [58]. Ammonia adsorption capacities were between 95.03 and 133.28 mg NH_3_/g. Competition experiments with methyl sulfide and dimethylsulfide showed excellent selectivity towards ammonia. To the best of our knowledge, the combination of imprinted polymer with g-C_3_N_4_ has not been reported for ammonia removal. 

### 4.4. Comparison to Conventional Adsorbents

Results in Figure 7 show that arsenate was removed similarly by the imprinted polymer with g-C_3_N_4_ (1.505 mg/g) and activated carbon (1.583 mg/g). The ion exchange resin Ambersep 21 K removed the most arsenate (1.852 mg/g). These results show that the material synthesized performs similarly to commercial materials used for the removal of arsenate. Regarding ammonia, the imprinted polymer with g-C_3_N_4_ removes the highest amount (0.271 mg/g) when compared to activated carbon (0.048 mg/g) and ion exchange resin Ambersep G-26-H (0.231 mg/g). Although the selectivity of the commercial materials has not been determined in this study, the imprinted polymers with g-C_3_N_4_ developed in this work performed to similar or higher capacities.

### 4.5. Initial Membrane Removal Results

The preparation method described provided membranes with a sufficient degree of structural integrity for testing. Results in Figure 8 show that arsenate was removed similarly by the non-imprinted and imprinted membrane (0.047 and 0.050 mg/g, respectively). However, in the competition experiments of the imprinted membrane with arsenate and chloride ions, arsenate binds more strongly to the membrane, selectively removing arsenate (0.053 mg/g) over chloride ions. The selectivity may be due to delayed permeation due to the arsenate binding in the imprinted site [59]. Ammonia removal was higher in the imprinted membrane (0.012 mg/g) but decreased in the competition experiment (0.011 mg/g), due to the lack of selectivity of the ammonia-imprinted polymer previously discussed.

## 5. Conclusions

Overall, this proof-of-concept study demonstrates that imprinted g-C_3_N_4_ adsorbents and membranes are effective in removing arsenate and ammonium ions from water. The imprinted polymers with g-C_3_N_4_ removed 1.505 mg of arsenate per gram of adsorbent and 0.271 mg of ammonium ion per gram of adsorbent. Moreover, in competition experiments, the arsenate-imprinted polymer with g-C_3_N_4_ showed excellent selectivity towards arsenate when compared to chloride ions. The ammonia-imprinted polymer with g-C_3_N_4_ did not show greater selectivity towards ammonium ions compared to calcium ions, probably due to higher calcium ion charge density and the g-C_3_N_4_ structure. While these novel materials have not been optimized, their adsorption capacity was comparable to commercial activated carbon and ion exchange resins for both arsenate and ammonium ion. 

Finally, when the materials were used to fabricate membranes, the membranes had sufficient structural integrity for testing. Arsenate and ammonium ion removal by the membranes followed the same trends as the column adsorption experiments. Further optimization will be performed on the materials to increase adsorption capacity, pressure stability, and structural integrity of the membranes, as well as antifouling properties and selectivity towards arsenate and ammonium ions.

## Figures and Tables

**Table 1:** Comparison between polymeric, ceramic and g-C_3_N_4_ membranes.

	Polymeric Membranes	Ceramic Membranes	g-C_3_N_4_ Membranes
**Permeability**	High permeability	High permeability [14]	High permeability [26]
**Selectivity**	Low selectivity [33,34]	Some selectivity [35]	High selectivity [24,36]
**Mechanical strength**	Good mechanical strength [37]	Prone to breakage [14]	Good mechanical strength [26]
**Pore size control**	Broader pore size distribution, smaller pore size [37]	Narrow pore size distribution, higher porosity, larger pore size [14,37]	Narrow pore size distribution, higher porosity [27,38,39]
**Fouling**	Susceptible to fouling [15]	Lower fouling [14,35]	Antibacterial and antifouling properties [26,27]
**Cleaning**	Susceptible to cleaning agents	Resistant to cleaning agents [14]	Self-cleaning properties [26,27]
**Integration with other processes**	Bioreactors [40]. Susceptible to oxidation reagents [39]	Advanced Oxidation Processes and bioreactors [39]	Advanced Oxidation Processes and adsorption [39]
**Thermal stability**	Poor [37]	Up to 500 °C [14,41]	Good [26]
**Cost of production**	Low cost [35]	High cost [14]	Expected low cost

**Table 2:** Arsenate removal in adsorption columns by different adsorbents with 20 mg/L influent arsenate concentration.

Adsorbent	Average Mass of Arsenate Removed/Mass of Adsorbent (mg/g)	Standard Deviation
Non-imprinted polymer	1.416	0.011
Imprinted polymer	1.412	0.100
Imprinted polymer competition	1.406	0.066
g-C_3_N_4_	1.583	0.011
Non-imprinted polymer + g-C_3_N_4_	1.469	0.180
Imprinted polymer + g-C_3_N_4_	1.505	0.078
Imprinted polymer + g-C_3_N_4_ competition	1.857	0.040

**Table 3:** Ammonia removal in adsorption columns by different adsorbents with 8 mg/L ammonia influent concentration.

Adsorbent	Average Mass of Ammonia Removed/Mass of Adsorbent (mg/g)	Standard Deviation
Non-imprinted polymer	0.070	0.012
Imprinted polymer	0.217	0.012
Imprinted polymer competition	0.068	0.003
g-C_3_N_4_	0.221	0.026
Non-imprinted polymer + g-C_3_N_4_	0.200	0.013
Imprinted polymer + g-C_3_N_4_	0.271	0.004
Imprinted polymer + g-C_3_N_4_ competition	0.057	0.013

## Data Availability

The data presented in this study are available within the article and Supplementary Materials.

## Supplementary Materials

The following supporting information can be downloaded at: https://www.mdpi.com/article/10.3390/polym14153146/s1, Figure S1: SEM images of non-imprinted materials for the removal of arsenate; Figure S2: SEM images of non-imprinted materials for the removal of ammonia. Figure S3. EDS mapping of arsenate imprinted membrane for the removal of arsenate.
