# Supplementary material for: Development of Adsorptive Membranes for Selective Removal of Contaminants in Water
Source: Polymers (Basel). 2022 Aug 2;14(15):3146. doi: 10.3390/polym14153146 (PMC9371136; doi:10.3390/polym14153146)
Supplement: Supplementary file 1 [file polymers-14-03146-s001.zip › polymers-1832614-supplementary.pdf]

Supplementary Materials

Development of adsorptive membranes for specific removal of contaminants in water

Priyalatha M. Kirisenage [1], Syed M. Zulqarnain [2], Jordan L. Myers [1], Bradley D. Fahlman [1], Anja Mueller [1], and Itzel Marquez [2,*]

[1] Department of Chemistry and Biochemistry, Central Michigan University, Mount Pleasant, MI 48859, USA;

[2] School of Engineering and Technology, Central Michigan University, Mount Pleasant, MI 48859, USA

*Correspondence: itzel1l@cmich.edu

A manuscript prepared for possible publication in

Polymers

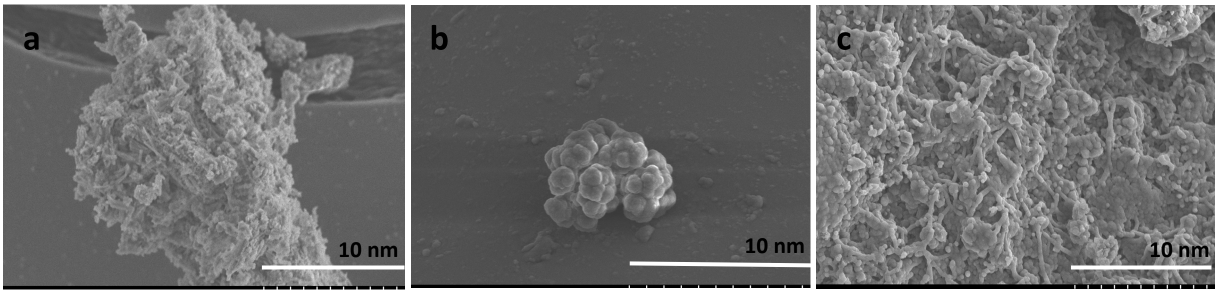

Figure S1. SEM images of non-imprinted materials for the removal of arsenate. (a) Non-imprinted polymer. (b) Non-imprinted polymer with g-C_3N_4. (c). Non-imprinted membrane

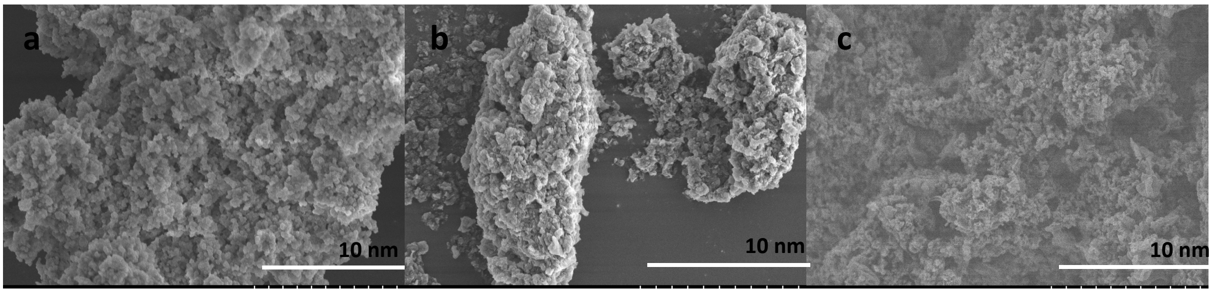

Figure S2. SEM images of non-imprinted materials for the removal of ammonia. (a) Non-imprinted polymer. (b) Non-imprinted polymer with g-C_3N_4. (c). Non-imprinted membrane

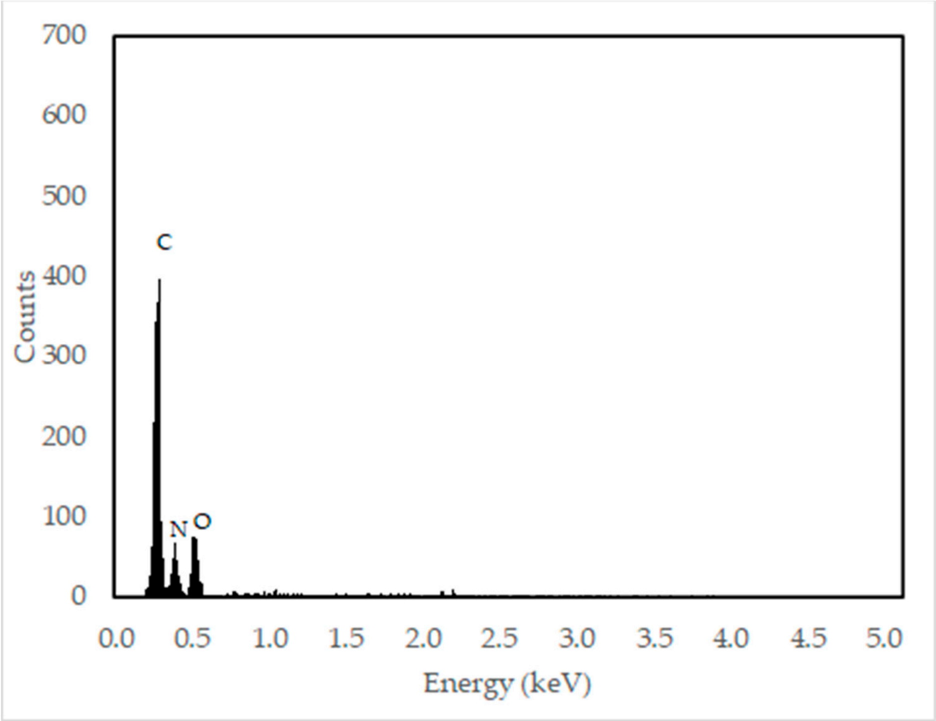

Figure S3. EDS mapping of arsenate imprinted membrane for the removal of arsenate. The arsenate concentration is below the detection limit of the instrument.
